# Synovial sarcoma of the hypopharynx – a case report and literature review^☆^^☆☆^

**DOI:** 10.1016/j.bjorl.2016.04.001

**Published:** 2016-04-26

**Authors:** Yasmine Kamhieh, Hannah Fox, Phillip Holland, Carl Passant

**Affiliations:** a Royal Gwent Hospital, ENT Department, Newport, United Kingdom; b Royal Gwent Hospital, Breast & Endocrine Surgery Department, Newport, United Kingdom

## Introduction

Synovial sarcoma of the head and neck is very rare, and sarcoma of the larynx and hypopharynx rarer still. As such, correct diagnosis and decision-making regarding surgery and adjuvant therapies pose clinical challenges to the otolaryngologist. We present a case of a synovial sarcoma arising from the hypopharynx, and a review of the literature.

## Case report

**Figure 1:**
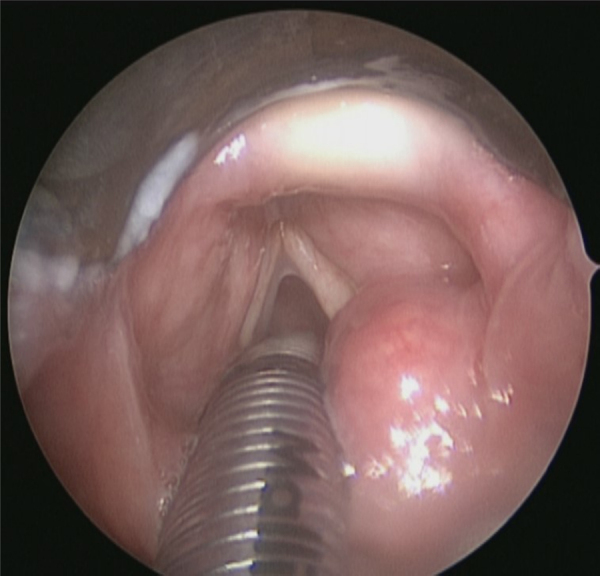
Panendoscopy view of a smooth lesion arising from the right arytenoid region.

A 77-year-old woman presented with intermittent hoarse voice and episodes of choking when swallowing. Panendoscopy showed a smooth, cystic mass arising from the right posterior aryepiglottic fold (Fig. 1). Initial biopsy showed only mild mucosal dysplasia. However, an urgent Computed Tomography (CT) scan was reported as showing a malignant-looking mass arising from the right superior thyroid pole. An ultrasound-guided biopsy showed spindle cells; differential diagnoses were a spindle cell variant of anaplastic thyroid carcinoma or Reidel's thyroiditis, but the sample was too small for definitive diagnosis.

**Figure 2:**
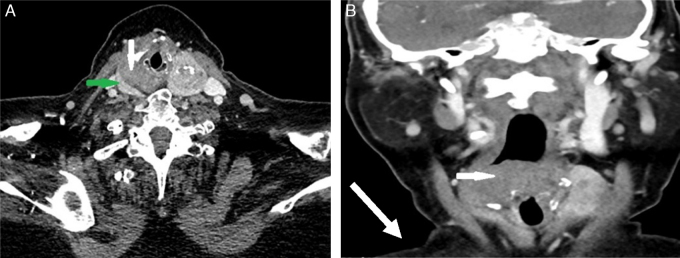
CT scan, with contrast, of the neck. (A) Axial view at C5/6 showing the enhancing right thyroid lobe (green arrow) and the non-enhancing mass arising from the hypopharynx (white arrow). A plane of separation is clearly visible in between. (B) Coronal view of the hypopharyngeal mass (white arrow).

The case was reviewed at the head and neck oncology MDT, consisting of two Radiology consultants, two Oncology consultants and four head and neck surgeons, as well as speech therapy and cancer nurse specialists. For this case we also invited input from the thyroid surgeon. It was felt that the quality of the first CT made it difficult to establish the origin of the mass; therefore the patient underwent a repeat CT. In these images, a visible tissue plane separated the mass and the right thyroid lobe (Fig. 2), indicating that the mass was in fact hypopharyngeal in origin. A further incision biopsy was performed under local anaesthetic and sent to the regional sarcoma panel. Fluorescence In Situ Hybridisation (FISH) analysis signal pattern was consistent with SS-18 gene rearrangement. The diagnosis was a synovial sarcoma of the hypopharynx.

Our patient had a background of idiopathic pulmonary fibrosis, with progressive dyspnoea on minimal exertion. She was offered curative surgery; however she repeatedly declined any therapy. She explained that she wanted to spend what time she had with her family, preserving the quality of her life as long as possible. With support from the palliative care team, she died at home, nine months after the confirmation of her diagnosis.

## Discussion

Synovial Sarcoma (SS) is a distinct malignant tumour of non-epithelial soft tissues. The name is a historical misnomer, as it is now recognised that they do not arise from joint synovia. It is most commonly seen as a slow-growing tumour in the extremities of adolescents and young adults; it is estimated that of approximately 200 presentations of SS per year in the U.K., 90% are in the extremities.^1^ They metastasise primarily haematogenously, to lung and bone. SS of the head and neck was first reported in the pharynx in 1954,^2^ but it remains a rare and poorly understood diagnosis.

On review of the English-language literature, we found 31 cases of laryngeal or hypopharyngeal SS (Table 1 shows a selection of reported cases). Median age was 21 years (range 11–79), with 72% male preponderance. The most common presenting symptoms were dysphagia (58%) followed by dysphonia (54%), similar presentations to other laryngopharyngeal malignancies.

**Table 1:** Selected reports of synovial sarcoma in the pharynx or larynx.

Author	Age	Presentation	Site	Treatment	Follow up
Jernstrom,^2^ 1954	21/M	Dyspnoea, dysphagia, neck lump	Hypopharynx	Died post laryngoscopy	N/a
Miller,^6^ 1974	23/F	Dyspnoea, dysphonia	Intra-arytenoid	Total laryngectomy	Disease-free 2 years
Gatti,^7^ 1975	23/F	Acute stridor	Intra-arytenoid	Supraglottic laryngectomy	Disease-free 2 years
Gatti,^7^ 1975	27/M	Dysphagia & dysphonia	Laryngopharyngeal	Partial laryngopharyngectomy	1 year – pulmonary metastases, Ch-RTX, died after 2.5 years
Fernandez-Acenero,^8^ 2009	12/M	Not documented	Supraglottic	WLE + ChTX	Disease-free 4 months
Bao,^9^ 2013	37/M	Sore throat, haemoptysis	Aryepiglottic fold	Partial laryngectomy + Ch-RTX	Died distant metastases 41 months
Yang,^10^ 2013	44/M	Dysphagia, dyspnoea	Hypopharynx	Total laryngectomy, pharyngoesophagectomy, neck dissection	Local recurrence 5 months – RTX, disease – free 2 years
Current case	77/F	Dysphonia + dysphagia	Arytenoid	Refused therapy	Died at 22 months

RTX, radiotherapy; ChTX, chemotherapy; Ch-RTX, chemoradiotherapy; WLE, wide local excision.

In the reported cases, imaging did not identify any consistent radiological features. In a review of the MRIs of 6 Head & Neck SS cases, Hirsch et al.^3^ reported the lesions were most commonly isointense to grey matter on T1 MRI, and may also be heterogeneous, haemorrhagic and multi-loculated. However Sigal et al.^4^ (3 cases) and Rangheard et al.^5^ (8 cases) did not identify consistent radiological features. In the reviews and the case reports, the tumour was usually reported to be well-circumscribed, unlike other malignancies. It is worth noting that in several of the cases, including ours, the clinical appearance of the tumours was also well-circumscribed and smooth, suggesting benign pathology.

The diagnosis in SS cases therefore rests heavily on the histology. The core biopsy fragments showed spindle cells; thin, elongated cells with scant cytoplasm. They are a non-specific finding, present in inflammatory tissue, benign masses and various neoplasms. SS may be “monophasic”, consisting solely of these spindle cells, or “biphasic”, consisting of spindle cells and epithelial elements. Immunohistology shows epithelial markers including cytokeratin and epithelial membrane antigen among others, and gene analysis yields t(X;18;p11;q11) translocation in over 90% of SS.^1^

Prognosis and management of these tumours has a limited evidence base. Of the 22 cases with a year or more of follow-up, 82% were disease-free at one year. Of the cases followed for 3 years, 67% were disease-free, 25% had died and one patient had a recurrence. 46% of the cases received primary surgery alone, 46% received surgery and adjuvant radiotherapy, and 7% received primary chemo-radiotherapy. Differing lengths of follow-up prevent meaningful comparisons of patient outcomes from the different therapeutic strategies.

## Conclusion

Synovial sarcoma of the head and neck is a rare but significant pathology. The lack of specific clinical and radiological features, and the smooth well-circumscribed lesions, can delay diagnosis and even recognition of their malignant nature. This case highlighted the importance of multi-disciplinary collaboration, and of early suspicion of sarcoma. This case, with those previously reported; serve to further clinical recognition of these rare but devastating tumours.

## Conflicts of interest

The authors declare no conflicts of interest.
